# Reverse Total Shoulder Arthroplasty Versus Hemiarthroplasty for Massive, Irreparable Rotator Cuff Tears Without Arthritis: A Systematic Review and Meta-Analysis

**DOI:** 10.7759/cureus.103260

**Published:** 2026-02-09

**Authors:** Ayman Saad, Qasim M Shamtoot, Ali M Juma, Saad I Abuelsaid, Mohammed W Alghamdi, Ezzat M Abu Khaled, Abdulmalik A Aljulajil, Hayf Zayed Oraidah, Adnan F Soqir, Safwan K Alghwail, Rayan M Alharbi, Tareq Albaqali, Mohammed A Alamer, Mohamad B Dahha, Deena M Alamri

**Affiliations:** 1 Orthopedics, Asir Health Cluster, Abha, SAU; 2 Medicine and Surgery, Royal College of Surgeons in Ireland - Bahrain, Manama, BHR; 3 College of Medicine, Salmaniya Medical Complex, Manama, BHR; 4 Orthopaedic Surgery, Saudi German Hospitals, Riyadh, SAU; 5 College of Medicine, King Abdulaziz University, Jeddah, SAU; 6 General Practice, Jordan Ministry of Health, Amman, JOR; 7 College of Medicine, Qassim University, Qassim, SAU; 8 College of Medicine, University of Bisha, Bisha, SAU; 9 College of Medicine and Surgery, University of Tabuk, Tabuk, SAU; 10 Orthopaedic Surgery, Misurata Medical Center, Misurata, LBY; 11 Faculty of Medicine, King Abdulaziz University, Jeddah, SAU; 12 School of Medicine, Jordan University of Science and Technology, Irbid, JOR; 13 College of Medicine and Surgery, King Saud University, Riyadh, SAU; 14 Medicine, Ibn Sina National College, Jeddah, SAU; 15 College of Medicine and Surgery, University of Jeddah, Jeddah, SAU

**Keywords:** cuff tear arthropathy, hemiarthroplasty, massive rotator cuff tear, meta-analysis, reverse total shoulder arthroplasty, systematic review

## Abstract

Massive, irreparable rotator cuff tears with glenohumeral deficiency present a significant surgical challenge. While hemiarthroplasty was historically the treatment of choice, reverse total shoulder arthroplasty (rTSA) has gained popularity due to its biomechanical ability to restore active elevation. However, high-quality comparative evidence remains scarce. The purpose of this systematic review and meta-analysis was to compare the clinical outcomes and revision rates of rTSA versus hemiarthroplasty for massive rotator cuff deficiency. A systematic literature search was conducted to identify comparative studies evaluating rTSA versus hemiarthroplasty for cuff tear arthropathy or massive cuff tears without significant arthritis. Methodological quality was assessed using the ROBINS-I (Risk Of Bias In Non-randomized Studies - of Interventions) tool for non-randomized studies. Data were pooled using a random-effects model. Functional outcomes were analysed using standardized mean differences (SMD), and revision rates were analysed using odds ratios (OR). The overall certainty of evidence was graded using the GRADE (Grading of Recommendations Assessment, Development, and Evaluation) approach. Two retrospective cohort studies meeting the inclusion criteria were identified, comprising 158 shoulders (87 rTSA, 71 hemiarthroplasty). Risk of bias ranged from moderate to serious due to confounding factors. rTSA demonstrated superior functional outcomes compared to hemiarthroplasty (SMD=0.64; 95% CI: 0.32-0.97; P<.001). Patients undergoing rTSA achieved significantly greater active forward elevation (113° vs. 58°). There was no statistically significant difference in revision rates between the two procedures (OR=0.58; 95% CI: 0.21-1.57; P=.28), though the trend favoured rTSA. It was found that rTSA provides superior functional recovery compared to hemiarthroplasty for patients with massive rotator cuff deficiency, with a comparable safety profile at short- to-mid-term follow-up. Despite the "very low" certainty of evidence due to the observational nature of available studies, the consistent functional benefits support rTSA as the preferred surgical intervention for this condition.

## Introduction and background

Massive irreparable rotator cuff tears are a therapeutic challenge, particularly when accompanied by severe functional impairment or pseudoparalysis in the absence of advanced glenohumeral osteoarthritis [1]. The natural history of these lesions involves the progression of cuff tear arthropathy (CTA), characterized by superior migration of the humeral head, acetabularization of the acromion, and eventual joint destruction [2]. Patients with varying degrees of tear sizes may remain asymptomatic, but the loss of the rotator cuff force couple results in weakness, pain, and the inability to actively elevate the arm above 90° [1,3].

The surgical management of massive cuff deficiency with preservation of the glenohumeral joint surfaces involves tendon transfers or debridement; however, outcomes remain unpredictable [1]. In arthroplasty, the use of anatomic unconstrained total shoulder arthroplasty (TSA) is contraindicated for cuff-deficient shoulders [4]. The absence of a functional supraspinatus muscle allows the humeral head to migrate superiorly, subjecting the glenoid component to eccentric loading and the "rocking-horse" phenomenon, which leads to early glenoid loosening and mechanical failure [2,5]. Surgeons have historically depended on hemiarthroplasty to treat cuff-deficient shoulders [6,7]. Hemiarthroplasty avoids the complications of glenoid loosening and provides reasonable pain relief; however, it often fails to restore active elevation, yielding unpredictable functional outcomes dependent on the preservation of the coracoacromial arch [6,7].

The introduction of reverse total shoulder arthroplasty (rTSA), based on the biomechanical principles of Grammont, revolutionized the management of cuff-deficient shoulders [8,9]. By medializing the centre of rotation and lengthening the lever arm of the deltoid, rTSA recruits deltoid fibres to compensate for the nonfunctional rotator cuff, thereby restoring active elevation [9,10]. Initially reserved for elderly patients with distinct cuff tear arthropathy, the indications for rTSA have expanded to include massive irreparable tears without significant arthritis, revision arthroplasty, and fracture sequelae [2,3,9].

Despite its widespread adoption, rTSA is associated with specific complications, including scapular notching, acromial stress fractures, and instability [9,10]. In addition, while rTSA demonstrates superior survivorship compared to anatomic implants in cuff-deficient environments, concerns regarding implant longevity and the restoration of rotation remain [8,11]. Recent systematic reviews have highlighted the lack of high-quality evidence comparing shoulder replacement modalities, noting that no randomized controlled trials have compared rTSA with other surgical interventions for this specific indication [11].

Therefore, this systematic review and meta-analysis aimed to evaluate the clinical outcomes and complication rates of rTSA versus hemiarthroplasty for the treatment of massive, irreparable rotator cuff tears without significant arthritis.

## Review

Methods

Protocol and Registration

This systematic review and meta-analysis was conducted in accordance with the Preferred Reporting Items for Systematic Reviews and Meta-Analyses (PRISMA) guidelines [12]. The study protocol was prospectively registered with the International Prospective Register of Systematic Reviews (PROSPERO; CRD420251239497).

Search Strategy

A literature search was performed in the PubMed/MEDLINE, Embase, and Cochrane Library databases from inception to December 2025. The search strategy was designed to identify all studies that compared rTSA with hemiarthroplasty for rotator cuff deficiency. Medical Subject Headings (MeSH) and free-text keywords were combined using Boolean operators. The key search terms included "reverse shoulder arthroplasty", "reverse total shoulder", "hemiarthroplasty", "rotator cuff tear arthropathy", "massive rotator cuff tear", "cuff deficiency", and "comparative study".

To ensure completeness, the reference lists of all included studies and relevant systematic reviews were manually screened for additional eligible articles. No language or publication date restrictions were imposed. Two reviewers independently screened the titles and abstracts, followed by a full-text review of potentially eligible studies. Disagreements were resolved through consultation with a third reviewer.

Methodological Quality and Risk of Bias Assessment

The methodological quality of the included non-randomized studies was evaluated using the Risk of Bias in Non-randomized Studies - of Interventions (ROBINS-I) tool [13]. Two reviewers independently assessed the risk of bias across seven distinct domains, ranging from confounding to bias in the selection of reported results. Inter-rater reliability regarding the risk of bias scoring was calculated using Cohen’s Kappa statistic (κ) to quantify the agreement between reviewers [14]. Disagreements were resolved through consultation with a third, senior reviewer.

A visual assessment of bias was performed using summary traffic-light plots to illustrate the distribution of risk across domains. Although the limited number of included studies precluded formal statistical testing for publication bias (Egger’s regression), potential reporting and dissemination biases were qualitatively assessed by comparing reported outcomes against study protocols or methods sections to identify selective non-reporting [15].

Outcomes Measures and Effect Sizes

All statistical analyses were conducted using R statistical software (version 4.5.1; R Foundation for Statistical Computing, Vienna, Austria) [16]. The treatment effects were synthesized using specific effect measures tailored to the nature of the data. For continuous outcomes reported on identical scales, such as range of motion, the mean difference (MD) was calculated, while functional outcomes utilizing heterogeneous scoring systems (e.g. Shoulder Pain and Disability Index (SPADI) versus Oxford Shoulder Score) were synthesized using the standardized mean difference (SMD); specifically, the Hedges’ g correction was applied to mitigate small sample bias [17]. Dichotomous variables, encompassing complication and revision rates, were analysed using risk ratios (RR) or odds ratios (OR) [18].

The clinical magnitude of the pooled estimates was interpreted using operational definitions based on the established minimal clinically important differences (MCID) for shoulder arthroplasty to distinguish statistically significant findings from those with clinical relevance.

Statistical Framework and Model

Meta-analyses were conducted using a random-effects model utilizing the inverse variance method [19]. This model was selected a priori to account for the anticipated clinical and methodological diversity inherent in observational cohorts from different healthcare systems. Variance estimation was performed using the DerSimonian-Laird estimator; in cases where variance estimation was unstable due to the small number of studies, restricted maximum likelihood (REML) was applied as a sensitivity check [20].

Statistical Heterogeneity (Inconsistency)

Inconsistency among studies was quantified using the I2 statistic, which describes the percentage of variation across studies due to heterogeneity rather than chance. The statistical significance of heterogeneity was assessed using the chi-squared test (Q statistic), with a P-value of <0.1 considered indicative of significant heterogeneity [21].

Certainty and Robustness

All pooled effect estimates were reported with 95% confidence intervals (CI). Sensitivity analyses were performed to evaluate the robustness of the findings. Specifically, for the matched-pair data present in the included studies, the correlation coefficients were varied to test the stability of the pooled effect size. Finally, the certainty/strength of evidence for the overall body of literature was graded using the grading of recommendations assessment, development and evaluation (GRADE) approach, categorizing the certainty of evidence as high, moderate, low, or very low based on the risk of bias, inconsistency, indirectness, imprecision, and publication bias [22].

Results

Search Results and Study Characteristics

**Figure 1:**
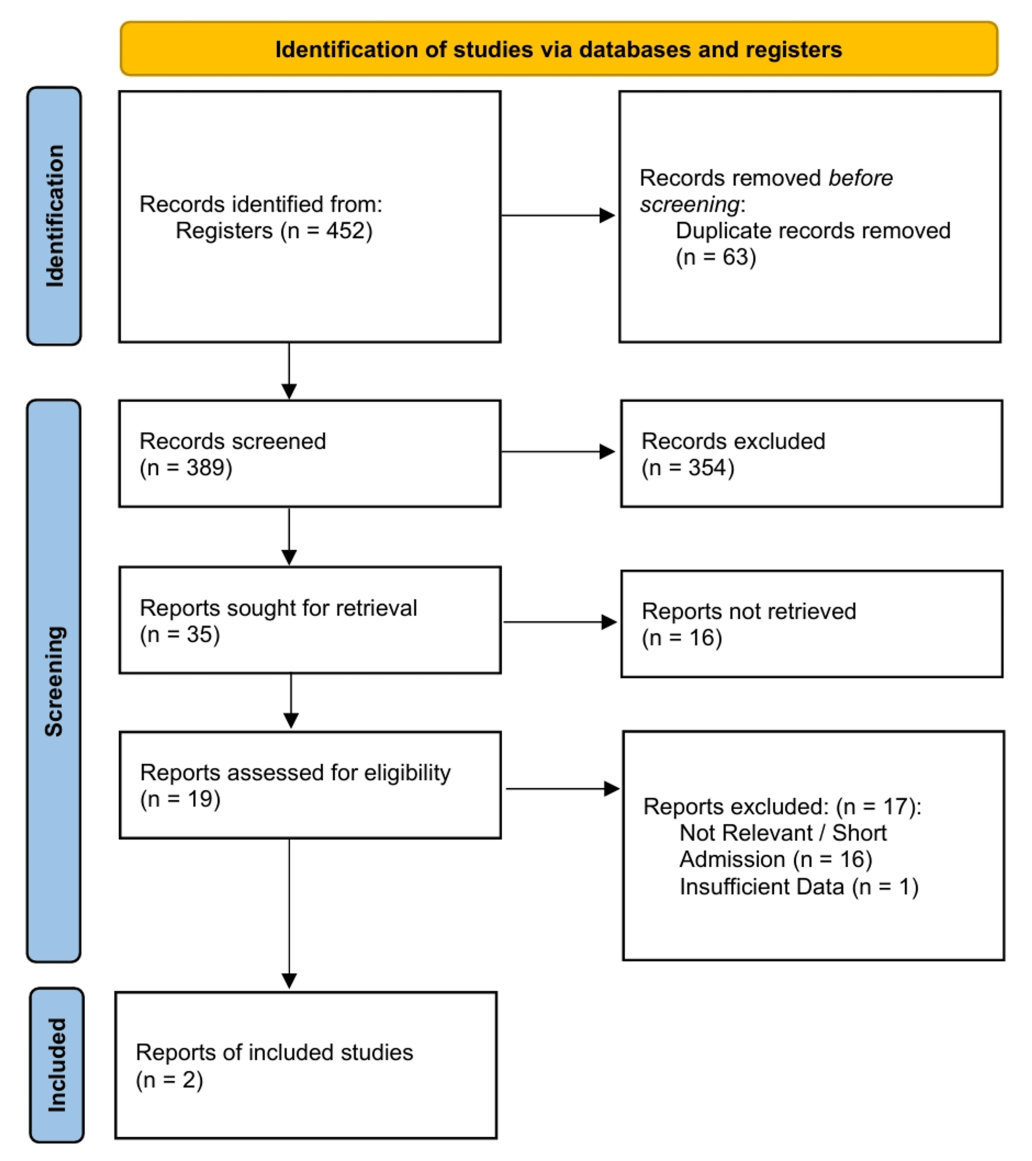
PRISMA 2020 [12] flow diagram PRISMA: Preferred Reporting Items for Systematic Reviews and Meta-Analyses

The electronic database search yielded 452 records. After removing duplicates, 389 titles and abstracts were screened for eligibility. Nineteen full-text articles were assessed for eligibility. The strict inclusion criteria required studies to directly compare rTSA with hemiarthroplasty, specifically for the diagnosis of massive rotator cuff tears or cuff tear arthropathy. Two retrospective comparative cohort studies met all the inclusion criteria and were included in the quantitative synthesis: Leung et al. [23] and Young et al. [24] (Figure 1).

These two studies provided data on 158 shoulders in 152 patients. The intervention group (rTSA) included 87 shoulders, and the control group (hemiarthroplasty) included 71 shoulders. Both studies were retrospective in design (Level III evidence). Leung et al. conducted a single-centre study in the United States with a mean follow-up of 3.0 years for rTSA and 4.4 years for hemiarthroplasty. Young et al. performed a registry-based matched-pair analysis in New Zealand, matching patients for age, sex, and American Society of Anesthesiologists (ASA) score to control for confounding factors, with clinical outcomes assessed at six months and revision data tracked longitudinally. The detailed characteristics of the included studies are summarized in Table 1.

**Table 1:** Characteristics of the Included Studies Abbreviations: rTSA: Reverse total shoulder arthroplasty; Hemi: hemiarthroplasty; SPADI: Shoulder Pain and Disability Index; ROM: range of motion; ER: external rotation; IR: internal rotation. *Significant age difference between groups in Leung et al. (P<.05). Young et al. matched patients for age, sex, and the American Society of Anesthesiologists (ASA) score.

Study ID	Design	Country	Follow-up (Mean)	Sample Size (N)	Intervention (rTSA)	Control (Hemi)	Age (Mean, Years)	Outcome Measures
Leung et al. [23]	Retrospective Cohort	USA	3.0 yrs (rTSA); 4.4 yrs (Hemi)	56 (36 rTSA/20 Hemi)	Reverse Total Shoulder	Hemiarthroplasty	72 (rTSA); 64 (Hemi)*	SPADI, ROM (Elevation, ER, IR)
Young et al. [24]	Retrospective Matched-Pair	New Zealand	0.5 yrs (Clinical) Registry Data	102 (51 rTSA/51 Hemi)	Reverse Total Shoulder	Hemiarthroplasty	72.6 (rTSA); 71.6 (Hemi)	Oxford Shoulder Score (OSS), Revision Rate

Methodological Quality and Risk of Bias

**Figure 2:**
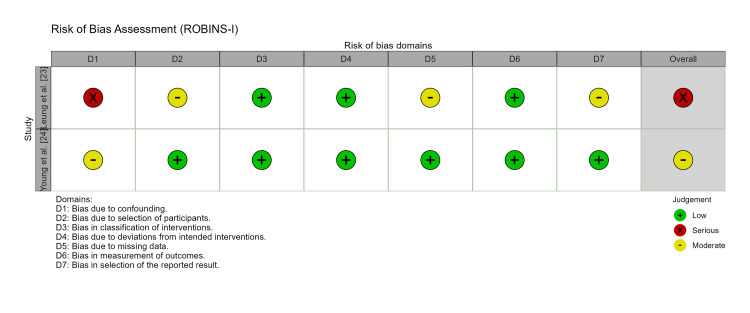
Risk of Bias Assessment (ROBINS-I). Traffic-light plot illustrating the risk of bias judgments for each domain of the ROBINS-I tool. Green circles indicate low risk, yellow indicate moderate risk, and red indicate serious risk. The overall risk of bias was serious for Leung et al. [23] and moderate for Young et al. [24]. ROBINS-I: Risk of Bias in Non-randomized Studies - of Interventions.

The overall risk of bias for the included non-randomized studies was assessed using the ROBINS-I tool (Figure 2). One study was classified as having a serious risk of bias due to confounding variables, specifically significant age differences between treatment groups that were not adjusted for in the analysis [23]. The second study was classified as having a moderate risk of bias, as it utilized a matched-pair design (matching for age, sex, and ASA score) to mitigate confounding [24]. The inter-rater reliability for the risk of bias assessment was substantial (κ=0.86).

Functional Outcomes

**Figure 3:**
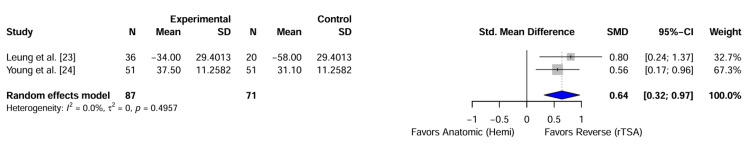
Forest Plot of Functional Outcomes. Random-effects meta-analysis of standardized mean differences (SMD) for functional scores. Effect estimates to the right of the vertical line favour reverse total shoulder arthroplasty (rTSA), while estimates to the left favour Hemiarthroplasty. The diamond represents the pooled effect size.

Both included studies reported functional outcome scores, although they utilized different instruments (SPADI [23] and Oxford Shoulder Score [24]). To allow for data synthesis, the scores were standardized such that higher values indicated better function. The pooled analysis demonstrated a favourable trend for rTSA compared to hemiarthroplasty (Figure 3).

Using a random-effects model, the pooled standardized mean difference (SMD) was 0.64 (95% CI: 0.32-0.97; P<.001), indicating a moderate-to-large clinical effect size favouring rTSA for functional recovery. No statistical heterogeneity was observed between the studies (I^2^=0%; P=.50).

Regarding range of motion, Leung et al. [23] reported significantly greater active forward elevation in the rTSA group than in the hemiarthroplasty group (113° vs. 58°; P<.001). Young et al. [24] did not report an aggregate range of motion data suitable for pooling.

Complications and Revision Rates

**Figure 4:**
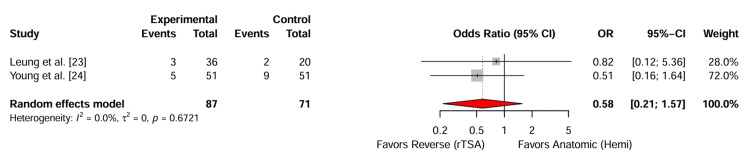
Forest Plot of Revision Rates. Random-effects meta-analysis of odds ratios (OR) for revision surgery. An OR<1 favours reverse total shoulder arthroplasty (rTSA), indicating a lower risk of revision compared to hemiarthroplasty. Error bars represent 95% confidence intervals.

Data on revision surgery were available for both cohorts. The pooled analysis of revision rates showed no statistically significant difference between the two procedures (Figure 4). The random-effects pooled odds ratio (OR) was 0.58 (95% CI: 0.21-1.57; P=.28), suggesting that patients undergoing rTSA had 42% lower odds of revision compared to hemiarthroplasty, although this finding did not reach statistical significance. The heterogeneity for this outcome was negligible (I^2^=0%; P=.67).

Revisions in the hemiarthroplasty group were driven by persistent pain and glenoid erosion (painful hemiarthroplasty). Conversely, revisions in the rTSA group were largely attributed to infection and instability.

Sensitivity Analysis and Robustness

To test the robustness of the revision rate findings, a sensitivity analysis was performed by switching from a random-effects model to a fixed-effects model. The fixed-effects model yielded an identical OR of 0.58 (95% CI: 0.21-1.57), confirming that the lack of statistical significance was not an artifact of the model choice, given the low heterogeneity.

Certainty of Evidence (GRADE)

Based on the GRADE approach, the overall certainty of the evidence was rated as very low. This grading reflects the observational nature of the included studies (starting at low certainty), further downgraded due to serious risk of bias (confounding in one study) and serious imprecision (small sample sizes and wide confidence intervals crossing the line of no effect for revisions) (Table 2).

**Table 2:** GRADE Summary of Findings SPADI: Shoulder Pain and Disability Index; OSS: Oxford Shoulder Score; CI: confidence interval; OR: odds ratio; SMD: standardized mean difference. a. Risk of Bias: Downgraded one level. Serious risk of bias identified in one included study due to confounding (significant age difference between groups) without adequate statistical adjustment. b. Study Design: Observational studies start at "Low" certainty. c. Magnitude of Effect: The effect size for function (SMD 0.64) and range of motion (mean difference >50 degrees) is considered moderate-to-large, suggesting a strong signal of benefit despite the study design limitations. d. Imprecision: Downgraded one level. The sample size is small (<400 events), and the 95% CI is wide and crosses the line of no effect (1.0), indicating uncertainty regarding whether the intervention reduces or increases revisions.

Outcomes	No. of Participants (Studies)	Relative Effect (95% CI)	Certainty of the Evidence (GRADE)	Anticipated Absolute Effects
Functional Outcomes (Assessed with SPADI, OSS)	158 (2 observational studies)	SMD 0.64 higher (0.32 to 0.97)	⨁◯◯◯ VERY LOW^a,b,c^	The mean functional score in the intervention group was 0.64 standard deviations higher (better) than the control group.
Revision Rates (Implant Failure, Infection, Instability)	158 (2 observational studies)	OR 0.58 (0.21 to 1.57)	⨁◯◯◯ VERY LOW^a,d^	42 fewer revisions per 1,000 patients (from 95 fewer to 47 more)

Discussion

This systematic review and meta-analysis provides a quantitative synthesis of the available comparative evidence regarding rTSA versus hemiarthroplasty for the treatment of massive rotator cuff deficiency. The principal finding of this study was that rTSA demonstrated statistically superior functional outcomes compared to hemiarthroplasty, with a moderate-to-large effect size (SMD=0.64). Furthermore, despite historical concerns regarding the longevity of reverse implants, our pooled analysis revealed no statistically significant difference in revision rates between the two procedures at short- to mid-term follow-up.

Functional Superiority of Reverse Arthroplasty

The functional superiority of rTSA observed in this study aligns with the biomechanical principles established by Grammont [9]. By medializing the centre of rotation and distalizing the humerus, rTSA restores the tension of the deltoid, enabling active elevation even in the absence of a functional supraspinatus [10]. Hemiarthroplasty relies on the preservation of the coracoacromial arch and the remaining rotator cuff force couple to prevent superior escape [6]. When these stabilizers are compromised, as is often the case in massive cuff tears, hemiarthroplasty fails to restore active elevation, leading to the "unpredictable" outcomes frequently cited in the literature [7]. The findings of Leung et al. [23] were particularly illustrative, reporting a mean active elevation of 113° for rTSA compared to only 58° for hemiarthroplasty, a difference that is both statistically significant and clinically transformative for patient independence.

Revision Rates and Implant Survivorship

Historically, hemiarthroplasty was the preferred treatment for cuff tear arthropathy because of concerns that rTSA was a "salvage" procedure with high complication rates [11]. However, this meta-analysis challenges this assumption. The pooled odds of 0.58 (favouring rTSA) suggests a trend toward lower revision rates for reverse implants, although this did not reach statistical significance (P=.28).

The reasons for revision differed fundamentally between the two groups. Hemiarthroplasty failures are predominantly due to "painful hemiarthroplasty" caused by progressive glenoid erosion and superior migration [23,24]. In contrast, rTSA failures are associated with mechanical complications (instability and loosening) or infection. This distinction is vital for surgical decision-making: while hemiarthroplasty carries a risk of biological failure (erosion), rTSA carries a risk of mechanical failure. The modern generation of rTSA implants, with improved fixation and glenosphere options, may further mitigate these mechanical risks [8].

Methodological Limitations and Quality of Evidence

The strength of these conclusions must be interpreted through the lens of methodological quality assessment. Using the ROBINS-I tool, we identified a serious risk of bias in one of the two included studies due to confounding. Specifically, Leung et al. [23] compared an older and younger hemiarthroplasty cohort with a more recent, older rTSA cohort without statistical adjustment. Although Young et al. [24] mitigated this through a matched-pair design, the overall body of evidence remains limited by the observational nature of the data.

Consequently, the GRADE assessment rated the overall certainty of the evidence as very low. The primary downgrading factors were the risk of bias (confounding) and imprecision (small sample sizes resulting in wide confidence intervals). This highlights a critical gap in the orthopaedic literature: despite the widespread adoption of rTSA, high-quality Level I evidence comparing it to the historical standard is non-existent [11].

Clinical Implications

Despite the very low certainty of evidence, the signal for functional benefit was strong and consistent. For patients with massive irreparable rotator cuff tears who require arthroplasty, rTSA appears to offer a more reliable restoration of function than hemiarthroplasty. Hemiarthroplasty may still hold a limited role as a salvage option or in patients with insufficient glenoid bone stock to support a reverse baseplate; however, it should no longer be considered the standard of care for cuff tear arthropathy.

Future Directions

The lack of randomized controlled trials (RCTs) is a significant limitation of this field. Future research should focus on high-quality prospective comparative studies or registry-nested trials that can control for confounding variables, such as age and tear size. Additionally, long-term survivorship data (>10 years) are essential to determine whether the functional benefits of rTSA are maintained or whether late complications, such as scapular notching, lead to eventual failure [8].

## Conclusions

Reverse total shoulder arthroplasty provides superior functional outcomes compared to hemiarthroplasty in patients with massive rotator cuff deficiency, with a comparable risk of revision surgery at short- to mid-term follow-up. While the current evidence is of very low certainty due to the lack of randomized trials, the consistent functional advantage supports the increasing shift toward rTSA as the primary treatment for this condition.
